# What Nurses Should Seek and Find

**Published:** 1918-04-20

**Authors:** 


### THE EDITOR'S LETTER-BOX.

### "What Nurses should Seek and Find."

*To the Editor of* The Hospital.

Sir,—My attention has just been drawn to an article in your issue of March 16, by "Ierne," on "Nursing Affairs in Ireland." I would not take any notice of what has been said, all of which the Irish Nurses' Association has been used to hearing from a very near and dear friend of "Ierne's" named "Shamus," except for the statement that the I.N.A. could and might effectually prevent a Registration Bill for Nurses becoming law. This suggestion is ridiculous, considering the fact that State Registration has for years been the main policy of the I.N.A. May I draw "Ierne's" attention to extracts from what his dear friend said in the *Weekly Irish Times* of March 25, 1916, regarding the I.N.A.?—"The I.N.A. is a thoroughly representative body. . . . Their views as to their own aims and desires are definite. In the foreground of their programme—burnt into it, in fact—is State Registration. For this they have been striving without ceasing for many years, and in their efforts they have the whole-hearted support of the rank and file. . . . The views of the I.N.A. . . . are very specific, very sound, and thoroughly democratic. . . . If they cannot obtain these (conditions), they will probably feel reluctantly compelled to stand aloof, and prefer to wait for a more convenient season to obtain the legislation, concerning which Irish M.P.s have expressed themselves favourably."

Let me assure "Ierne" or "Shamus" that is exactly what the "dames" of the I.N.A., in their "parlour in Stephen's Green," are now doing. They accomplished a good piece of work lately in having delayed the passage of the Midwives Bill for Ireland long enough to secure seats on the Central Council for *four* midwives, instead of *one* as originally arranged.—I am, Sir, yours, etc.,

A Member of the I.N.A.

Dublin, April 6.

[Camouflage! Encore camouflage! Toujours camouflage! In March 1916 there was only one Association of Nurses in Ireland. Since that date the camp divided, one party adopting the policy of forming an Imperial Union of Nurses; the other, following Sinn Fein tactics, declared for "ourselves alone." These latter are the dames of Stephen's Green, who have formed an Irish Board of Nurses, which the Royal College of Physicians decline to recognise. If they claim to represent Ireland, the proof is easy. Nothing is more convincing than a statement of revenue from members' subscriptions with names.

The concluding paragraph demonstrates my contention as to the mischief which these women may work. I have it on the highest authority that their blocking of the Midwives Bill almost caused its withdrawal. It was only under great pressure, and on the assurance that it was non-contentious, that the Government consented to permit its introduction during the war. The Scottish model was accordingly followed. But the moment it appeared they assembled in their parlour, and circularised Irish M.P.s requesting them to hold it up for amendment. No matter how small their number or their membership, they can still pursue such a policy of obstruction.—Ierne.]

